# Analysis of effect of congenic mice on the gene expression under genomic background of BALB/c mice

**DOI:** 10.1186/1471-2105-15-S10-P10

**Published:** 2014-09-29

**Authors:** Yan Jiao, Xiaoyun Liu, Yanhong Cao, Nan Deng, Yonghui Ma, Karen A Hasty, John M Stuart, Weikuan Gu

**Affiliations:** 1 Mudanjiang Medical College, Mudanjiang, 157001, PR China; 2 Departments of Orthopaedic Surgery and Biomedical Engineering, University of Tennessee Health Science Center, Memphis, TN 38163, USA; 3 Institute of Kaschin-beck Disease, Center for Endemic Disease Control, Chinese Center for Disease Control and Prevention, Harbin Medical University; Key Laboratory of Etiologic Epidemiology, Education Bureau of Heilongjiang Province & Ministry of Health (23618104), Harbin, 150081, China; 4 Department of Medicine, University of Tennessee Health Science Center, Memphis, TN 38163, USA; 5 Research Service, Veterans Affairs Medical Center, 1030 Jefferson Avenue, Memphis TN 38104, USA

## Background

Interleukin 1 (IL-1) is a major contributor to the development of immune-mediated arthritis. BALB/c mice that are homozygous for the deficiency (BALB/c^-/-^) spontaneously develop joint-specific inflammation that resembles human rheumatoid arthritis [1]. To understand the role of genetic factors involved in the development of spontaneous arthritis (SAD) in mice deficient in IL-1 receptor antagonist protein (IL_1RA), we have identified a genomic region containing a major quantitative trait locus (QTL) for this disease. The QTL is on chromosome 1 and appears to be the strongest genetic region regulating arthritis [2]. To confirm the importance of this QTL in SAD, we next developed congenic mouse strains that contain the strong QTL genomic fragments from BALB/c on a DBA/1 genetic background [3] and conversely a similar genomic fragment from DBA/1 on a BALB/c background. When the DBA/1 fragment was placed on a BALB/c background, arthritis was delayed and less severe. When the BALB/c fragment was placed on a DBA/1 background, arthritis occurred in varying degrees.

## Materials and methods

To further narrow down the list of potential candidate genes, we analyzed gene expression profiling on mouse whole genome scale using microarray technology. Female mice at 4 months of age from a congenic strain BALB.D1-1^-/-^ and BALB/c^-/-^-mice were used to generate gene expression data using the Illumina platform. The experiment for each strain was done with three replicates, each from one female mouse. Raw data were normalized with the quantile method using BeadStudio software. The DiffScore in our initial analysis was set up as ± 10, to ensure we did not miss the potential candidate genes.

## Results

Our analysis of gene expression successfully produced extremely useful information. Two very important features of the result are that 1) a very small number of genes is differentially expressed between BALB.D1-1-/- and BALB/c-/-, and 2) a majority of the differentially expressed genes are located in the QTL region. There are only 12 probes that are differentially expressed between BALB.D1-1-/- and BALB/c-/-. Among the 12 differentially expressed probes, five represent known genes (Rnpep, Ifi203, Lefty1, Trbv6, Ifi202b) and three sequences are similar to Ifi genes (interferon-activatable genes, similar to Ifi204, and similar to Ifi205, Figure 9a). Among them, six probes represent genes within the QTL (Ifi203, Lefty1, Ifi202b, interferon-activatable genes, similar to Ifi204, similar to Ifi205).

## Conclusions

Several important candidate genes in the molecular pathways of SAD have been identified from the mouse model.
